# Vaginal practices among women at risk for HIV acquisition in Soweto, South Africa

**DOI:** 10.4102/sajhivmed.v20i1.866

**Published:** 2019-06-20

**Authors:** Erica Lazarus, Kennedy Otwombe, Janan Dietrich, Michele P. Andrasik, Cecilia A. Morgan, James G. Kublin, Glenda E. Gray, Abby J. Isaacs, Fatima Laher

**Affiliations:** 1 Perinatal HIV Research Unit, Faculty of Health Sciences, University of the Witwatersrand, Johannesburg, South Africa; 2 HIV Vaccine Trials Network, Fred Hutchinson Cancer Research Center, Seattle, United States; 3 South African Medical Research Council, Cape Town, South Africa; 4 Statistical Center for HIV/AIDS Research and Prevention, Seattle, United States

**Keywords:** Vaginal hygiene, Vaginal practices, HIV risk, Young women, Soweto, South Africa

## Abstract

**Background:**

Vaginal practices (VP) may adversely affect normal vaginal flora and mucosal integrity, and increase acquisition risk of HIV and other genital tract infections.

**Objective:**

The aim of this study was to describe self-reported VP, changes in the reported number of VP over time and factors associated with VP in a cohort of young Sowetan women enrolled in the HVTN 915 observational study.

**Method:**

We longitudinally assessed self-reported VP in 50 young women at risk of HIV acquisition aged 18–25 years in a prospective study over 3 months in Soweto, South Africa. Interviewer-administered HIV behavioural risk questionnaires were completed. No intervention to reduce VP was specified per protocol, but clinicians provided education at their discretion. The generalised estimating equation with inverse probability weights assessed VP over time.

**Results:**

The mean age at screening was 22 years; women reported multiple sexual partnerships with a mean of one main and 2 casual partners in the last 30 days. Consistent condom use was 2% (*n* = 1), 25% (*n* = 12) and 43% (*n* = 3) with main, casual and new partners, respectively. Commonly reported VP included washing the vagina with water (44%) and using fingers (48%). VP decreased significantly over time (*p* < 0.001). Women who used condoms inconsistently or whose last sex was with a casual partner were 3 times more likely to report VP (*p* = 0.001).

**Conclusion:**

Despite the high incidence of HIV in our setting, VP are still common and are associated with other behavioural risks for HIV. Further study is needed to assess whether clinician education may reduce VP and therefore should be included in HIV risk reduction counselling.

## Introduction

In 2016, an estimated 17 million women aged 15 years and older were living with HIV.^1^ In Eastern and Southern Africa, women aged 15–24 years accounted for 26% of new infections.^2^ In South Africa, the sub-Saharan African country with the highest number of people living with HIV, HIV prevalence among 20–24-year-olds was 16%.^3^

Young women in South Africa are twice as likely to acquire HIV compared with their male peers.^3^ Various biological, social and behavioural factors increase the susceptibility of women to HIV acquisition.^4,5^ Vaginal practices (VP) have been shown to increase the acquisition risks of HIV in women.^6^ While all intravaginal washing increased the risk of HIV, women who washed intravaginally with soap or other substances were at higher risk than those who washed with water alone.^6^ In addition, VP increased the risk of other genital tract infections (GTIs) that themselves are associated with increased HIV acquisition risk, including Herpes Simplex Virus type 2,^7^ Human Papillomavirus^8^ and bacterial vaginosis.^9,10,11,12,13^ Furthermore, VP may also reduce the effectiveness of HIV prevention methods like microbicides.^14^

Vaginal practices (VP) include a range of practices, such as washing the external genitalia, vaginal washing with water alone or with water and soap,^8,9,12,15,16,17,18,19,20,21,22^ which may be performed with or without a cloth.^10,13,23,24^ In addition, insertion of substances for enhancement of sexual pleasure, particularly vaginal drying, has also been reported by women from as young as 16 years of age.^9,21,25,26^

Vaginal practices are commonly self-reported by women in African communities.^13^ Among female sex workers in Kenya, 86% – 100% of women report VP.^10,12,19,20,27^ In the KwaZulu-Natal Province of South Africa, a household survey found that 90% of women practised some form of VP,^28^ whereas data from Cape Town in the Western Cape Province reflects much lower practice, around 26% – 29%.^23,24^

We were unable to find any study in the literature reporting on whether partner type has an effect on VP. However, Scorgie et al. reported that women in KwaZulu-Natal, who were in stable relationships but also had other partners, were significantly more likely to use VP for sexual motivations than women who did not have casual partners.^29^

Furthermore, there are limited longitudinal data on the change in VP over time among South African women. Only one study including South African women assessed, and counselled against, VP at all study visits.^30^ In this study, quarterly counselling decreased vaginal washing practices by less than 10%.^30^

We describe self-reported VP in a cohort of young Sowetan women enrolled in the HVTN 915 observational study. In addition, we assess the change in the reported number of VP over time as well as factors associated with VP, including casual sexual partnerships.

## Methods

### Study design

HVTN 915 was a prospective observational study of 50 women deemed to be at risk of HIV acquisition, in Soweto, South Africa. The study was conducted between August 2014 and April 2015, and aimed to evaluate the feasibility of self-administered vaginal swabs for the detection of HIV-1 virions transferred through vaginal sexual intercourse and to compare sexual and behavioural risk data collected via in-person interview versus daily mobile phone survey. In this article, we focus on the VP reported by women in HVTN 915.

### Study setting

The study was conducted at the Perinatal HIV Research Unit in Soweto, South Africa. Soweto, with a population of about 1 million people, is located south-west of Johannesburg in the Gauteng Province of South Africa. The HIV prevalence in adults in Gauteng is 18%.^3^ Women were recruited from surrounding areas, including local taverns, and invited to come to the site to receive more information about the study.

### Participants

Women were eligible for participation in the study if they were between the ages of 18 and 25 years, healthy on the basis of medical history and physical examination and HIV-uninfected. Epidemiological data on HIV prevalence in South Africa guided the eligibility criteria for women who may be at risk of HIV acquisition: (1) women who had vaginal intercourse with one or more males 4 or more times per week in the 30 days preceding screening; (2) black African women aged 20–24 years; (3) women who engaged in transactional sex; (4) unmarried women living together with a partner; (5) women who engaged in unprotected vaginal or anal sex (sex without a condom); (6) women who lived or worked in informal settlements and taverns, and (7) women with any history of genital ulcer disease.^31^ Volunteers for the study provided written informed consent for study procedures and data collection prior to beginning screening procedures. Women were excluded if they were pregnant or breastfeeding, or if they were unwilling to use contraception for the duration of the study. Mandated contraceptives were condoms, diaphragm or cervical cap, intrauterine device, hormonal contraception, vasectomy of male partners or no reproductive potential such as hysterectomy. Women who became pregnant during the study were terminated from study follow-up and referred for antenatal care at local health facilities. Women who became infected with HIV during the study were to be referred to local health facilities for care, but could continue in the study.

### Procedures

Eligible participants attended 9 study visits over 3 months. To fulfil the primary objectives of the study (not reported in this article), participants self-collected a daily vaginal swab. At each study visit, participants answered a quantitative interviewer-administered, pen-and-paper-based HIV behavioural risk questionnaire. Recall period at screening was 30 days and 7 days at subsequent visits. The questionnaire included questions on sexual behaviour, alcohol and drug use, and VP. VP were assessed through 12 items addressing vaginal washing, insertion of medicines or other items (paper, cloth, sponges, cotton wool, tampon) and use of gels, lubricants or creams, including haemorrhoid creams. These items were derived from the literature and are in line with the WHO policy brief on gender, sexuality and VP.^22^ Participants were asked to report the frequency of each practice during the recall period: never, once a week, 2–3 times per week, 4–5 times per week or every day. Staff were trained on the administration of the questionnaire and the use of interviewer cards to remind participants of the frequency options.

Participants received individualised HIV risk reduction counselling at every visit and HIV counselling and testing at screening, and weeks 6 and 9. In addition, participants were assessed for GTIs at enrolment by screening for symptoms and signs of GTIs as well as laboratory testing. Symptom screening and treatment for GTI syndromes were performed using the South African standard of care syndrome-based approach.^32^ The following GTI syndromes were assessed: lower abdominal pain, vaginal discharge syndrome, genital ulcer syndrome, bubo, syphilis, pubic lice, genital warts and any other genital tract symptoms. In addition, a Pap smear, blood test for syphilis and urine polymerase chain reaction for *Chlamydia trachomatis* and *Neisseria gonorrhoea* were collected on all participants. Where vaginal discharge was reported or found on speculum examination, pH testing, microscopic examination of wet mount specimen and whiff test for bacterial vaginosis and OSOM^®^ Trichomonas test for *Trichomonas vaginalis* were also performed.

No intervention to reduce VP was mandated in the protocol, but at their discretion, clinicians discussed the topic with participants as part of health education that was not required to be documented.

### Statistical analysis

Statistical analysis was conducted using SAS Enterprise Guide 6.1 (SAS Institute, Cary, NC). Baseline measures at screening were assessed descriptively for the main, casual and new partners. Continuous measures such as age of participant, age of sexual partners and number of sexual partners were assessed using means, standard deviations (s.d.) and ranges; frequencies and their percentages were determined for categorical measures such as frequency of condom use (categorised as inconsistent if reported as ‘never’ or ‘sometimes’ vs. consistent if reported as ‘always’) and alcohol use. Condom use was not specifically categorised by transactional versus non-transactional sex acts.

Frequencies were similarly determined for VP at screening and the final visit at month 3. Vaginal practices were classified as ‘ever’ or ‘none’ for each recall period, where ‘ever’ referred to any report of the VP in question regardless of frequency and ‘none’ was defined as never having engaged in that VP. VP, risk behaviours including number of sexual partners, frequency of condom use, transactional sex, and alcohol and drug use were compared at screening and final visit using McNemar’s test. The laboratory GTI test results and symptomatic GTIs at enrolment were similarly compared.

The pattern of missing data at each follow-up visit was assessed to determine whether it was monotone or intermittent. A weighted generalised estimating equation (GEE) was used to model enrolment factors associated with VP during follow-up where the dependent variable was an indicator of VP at each visit. Variables from the behavioural risk assessment at enrolment were used as covariates in the modelling. These included participant age, having a main sexual partner, age of the main partner, having casual partners, frequency of vaginal sex, consistent or inconsistent condom use with any of the partners, transactional sex, whom they last had sex with among the partner types and laboratory-confirmed GTI results. The univariate GEE model was fitted followed by the multivariate model. Modelling assumed a compound symmetry covariance structure, a binomial distribution for the dependent variable and a logit link. All the variables with a *p*-value of < 0.1 at the univariate level were considered for entry into the multivariate model followed by application of a stepwise selection procedure.

### Ethical consideration

The study was approved by the University of the Witwatersrand Human Research Ethics Committee (Ethics reference number: 131114).

## Results

### Demographics and sexual behaviour

There were 50 women enrolled, with a mean age of 22 years (s.d. 2.0). At screening, women reported multiple sexual partnerships (Table 1), with a mean of one main partner for 49 women and 2 casual partners for 48 women in the last 30 days. Seven women reported one new partner in the last 30 days. The mean ages of main, casual and new partners were similar (27.0, 26.0 and 26.8 years, respectively). The average number of times women had sex in the last 30 days was different by partner type: 15.3 times with their main partner, 10 times with their casual partner and 3.6 times with a new partner. Women reported the nature of their partnerships to be transactional in 14.3% (*n* = 7) of main, 41.7% (*n* = 20) of casual and 66.7% (*n* = 4) of new partnerships. Although 59.6% of women reported that their casual partners were having sex outside of their partnership, 73.5% of women reported that they did not know if their main partners had other sexual partners.

**TABLE 1:** Screening sexual risk and substance use behaviours (in the last 30 days).

Sexual risk behaviour variable by partner type	Main partner (*n* = 49)			Casual partner (*n* = 48)			New partner (*n* = 7)^†^		
	*N*	%	s.d.	*N*	%	s.d.	*N*	%	s.d.
**Number of sexual partners (vaginal or anal)**									
Mean	1	-	0.3	2	-	0.8	1	-	0.0
Range	1–3	-	-	1–4	-	-	1–1	-	-
**Age of sexual partner (years)**									
Mean age of partner	27.0	-	3.3	26.0	-	4.6	26.8	-	3.9^†^
Range	21–35	-	-	17–37	-	-	22–32	-	-
**Number vaginal sex acts**									
Mean	15.3	-	7.7	10.0	-	8.6	3.6	-	2.0
Range	3–31	-	-	1–38	-	-	1–7	-	-
**Frequency of condom use**									
Never	44	89.8	-	12	25.0	-	2	28.6	-
Sometimes	4	8.2	-	24	50.0	-	2	28.6	-
Always	1	2.0	-	12	25.0	-	3	42.9	-
**Transactional sex**									
Yes	7	14.3	-	20	41.7	-	4	66.7	-
No	42	85.7	-	28	58.3	-	2	33.3	-
**Partner having other concurrent vaginal or anal sex partners**									
Yes	10	20.4	-	28	59.6	-	3	42.9	-
No	3	6.1	-	0	0	-	0	0	-
Don’t know	36	73.5	-	19	40.4	-	4	57.1	-
**Substance use behaviour**									
**Alcohol use**									
Yes	39	78	-	-	-	-	-	-	-
No	11	22	-	-	-	-	-	-	-
**How often did you drink alcohol?**									
Less than once a week	13	33	-	-	-	-	-	-	-
1–2 times a week	18	46	-	-	-	-	-	-	-
3–4 times a week	8	21	-	-	-	-	-	-	-
**On the days that you drink alcohol, how many drinks do you usually have?**									
2–3 drinks	1	3	-	-	-	-	-	-	-
4–5 drinks	9	23	-	-	-	-	-	-	-
6 or more drinks	29	74	-	-	-	-	-	-	-
**Did you inject any non-prescription drugs?**									
Yes	0	0	-	-	-	-	-	-	-
No	50	100	-	-	-	-	-	-	-
**Did you take any non-injected drugs?**									
Yes	20	40	-	-	-	-	-	-	-
No	30	60	-	-	-	-	-	-	-
**How often do you use cigarettes or tobacco products?**									
Never	30	60	-	-	-	-	-	-	-
Once a week	1	2	-	-	-	-	-	-	-
Every day	16	32	-	-	-	-	-	-	-
2–3 times a week	2	4	-	-	-	-	-	-	-
4–5 times a week	1	2	-	-	-	-	-	-	-

s.d., standard deviation.

† , One participant had no entry for age of new sexual partner or transactional sex, but had entries for number of new partners, number of times they had vaginal sex, condom use and partner’s concurrent partners.

The proportion of women who reported always using condoms also varied by partner type: 2.0% (*n* = 1) with main partner, 25.0% (*n* = 12) with casual partners and 42.9% (*n* = 3) with new partners. However, condom use increased by the last visit with 20.0% (*n* = 7/35) of women using condoms with their main partner and 56.0% (*n* = 9/16) in casual partnerships.

During follow-up, one participant was withdrawn because of non-adherence to all protocol procedures, one participant was terminated from the study because of pregnancy and 2 participants were lost to follow-up. Missing data occurred intermittently at weeks 1 (*n* = 1, 2%), 3 (*n* = 3, 6%), 4 (*n* = 1, 2%), 6 (*n* = 3, 6%), 8 (*n* = 3, 6%) and 12 (*n* = 4, 8%). There were no incident HIV infections during the study.

### Vaginal practices

Table 2 shows the VP at screening: washing vigorously outside the vagina (10%), washing with water (44%) or something else (32%), using fingers (48%) or something else (28%) to wash inside the vagina, placing traditional medications inside the vagina (2%) and using tampons (8%). Although specifically asked, no women reported using conventional medications, paper, cloth, sponges, cotton wool, gel, lubricants, creams including haemorrhoid creams or anything else inside the vagina. Compared to screening, at month 3 there were significant declines in women reporting washing inside the vagina with water (*p* < 0.001) or with something else besides water (*p* < 0.001) as well as significant reductions in the use of fingers (*p* < 0.001) or other means (*p* < 0.001) to wash inside the vagina (Table 2).

**TABLE 2:** Vaginal practices at screening^†^ and at month 3.

Vaginal practices^‡^	Screening		Month 3		*p*
	*n*	%	*n*	%	
**Number of respondents**	**50**	**-**	**46**		**-**
Washed vigorously the outside of vagina	5	10.0	5	10.9	1.00
Washed with water inside vagina	22	44.0	6	13.0	< 0.001
Washed with something else besides water inside vagina	16	32.0	2	4.0	< 0.001
Used fingers to wash inside vagina	24	48.0	2	4.3	< 0.0001
Used something else to wash inside vagina	14	28.0	1	2.2	< 0.001
Put or kept traditional medicines inside vagina	1	2.0	0	0	n/a
Used a tampon	4	8.0	1	2.0	0.08
Any vaginal practice	21	42.0	8	17.4	0.0143

n/a, not applicable.

† , Screening recall period: last 30 days; month 3 recall period: last 7 days.

‡ , Participants reporting engaging in the practice once a week, 2–3 times per week, 4–5 times per week or every day.

Overall, each visit was associated with a drop of 0.47 (*p* < 0.001) in the number of participants reporting VP.

### Genital tract infections

On the laboratory GTI tests conducted at enrolment, 20 (40%) participants had a positive result with 5 (10%) having more than one infection concurrently (4 had two infections and 1 had 3 infections): 4 (8%) had gonorrhoea, 12 (24%) *C. trachomatis*, 1 (2%) trichomoniasis and 7 had markers of bacterial vaginosis (6 [12%] positive pH and 1 [2%] had both a positive pH and whiff test). Only 9 (18%) women reported a symptomatic GTI at enrolment. All 9 reported vaginal discharge and none reported lower abdominal pain, genital ulcer syndromes or other GTIs. The difference between laboratory-confirmed GTIs and symptomatic GTIs was significant (*p* = 0.0184).

### Factors associated with vaginal practices

As shown in Table 3, women who reported inconsistent use of condoms at enrolment compared to consistent condom use (odds ratio [OR]: 3.39, 95% confidence interval [CI]: 1.70–8.78) and those who last had sex with a casual partner prior to enrolment versus a main partner (OR: 3.03, 95% CI: 1.56–5.88) had a higher odds of reporting VP. The presence of a laboratory-confirmed GTI was not associated with VP over time.

**TABLE 3:** Factors associated with vaginal practices during follow-up.

Variable	Univariate					Multivariate		
	*N* or median	% or IQR	OR	95% CI	*p*	OR	95% CI	*p*
Participant age (years) at enrolment	22	21–24	1.0535	0.8933–1.2426	0.5357	-	-	-
**Do you have any main sexual partners?**								
Yes	49	98%	Ref.	-	-	-	-	-
No	1	2%	7.8729	5.5694–11.1291	<0.0001	-	-	-
Age (years) of main partner	27	24–29	1.0007	0.9092–1.1014	0.9883	-	-	-
**Do you have any casual sexual partners?**								
Yes	48	96%	4.5461	1.0118–20.4254	0.0482	-	-	-
No	2	4%	Ref	-	-	-	-	-
In the last 30 days, how many times have you had vaginal sex with your main and/or casual and/or new partner?	3	2–3	0.9904	0.9664–1.0150	0.4421	-	-	-
**Condom use in the last 30 days, during vaginal sex with your main and/or casual and/or new partner**								
Consistently	12	24%	Ref.	-	-	Ref.	-	-
Inconsistently	38	76%	3.4086	1.5306–7.5906	0.0027	3.3860	1.6980–8.7748	0.0013
**Transactional sex in the last 30 days with your main and/or casual and/or new partner**								
Yes	21	42%	1.1635	0.5723–2.3656	0.6757	-	-	-
No	29	58%	Ref.	-	-	-	-	-
**The last time you had sex, whom did you have sex with?**								
Casual partner	6	12%	2.5242	1.0593–6.0154	0.0366	3.0285	1.5610–5.8756	0.0010
Main partner	44	88%	Ref.	-	-	Ref.	-	-
**Genital tract infection (test results)**								
Positive	19	38%	0.9189	0.4490–1.8804	0.8169	-	-	-
Negative	31	62%	-	-	-	-	-	-

IQR, interquartile range; OR, odds ratio; CI, confidence interval.

## Discussion

Our study shows that VP are common among Sowetan women at risk of HIV acquisition, but can be reduced within a relatively short period of time. In addition, we show that sex with a casual partner increases the odds of vaginal hygiene practices.

Although our study did not explore motivations, vaginal hygiene is the most common reported reason for VP used by both African and non-African women alike. A cross-sectional study in Cape Town, South Africa, which enrolled 2897 women, found that 29% used VP, 53% of whom did so for regular hygiene purposes.^23^ In Tanzania, more than 99% of women reporting VP did so for hygiene reasons.^16^ Other common reasons for VP include pre- or post-coitally for hygiene or to enhance sexual pleasure.^17,25,26,33,34^

Half the women in our study reported engaging in some form of VP, most commonly washing with water and with fingers inside the vagina. This is similar to other studies in high-risk women in sub-Saharan Africa, including other regions of South Africa. In Gambia, Zambia and Tanzania, VP are considered part of routine hygiene.^16,17,35^ In KwaZulu-Natal Province of South Africa, VP were shown to be a learned or cultural practice passed down in families.^29,36^ This may also be the case in Sowetan women. Another exacerbating factor might be the influence of increased advertising and availability of feminine hygiene products for genital cleansing to women in urban areas like Soweto. These products are promoted as having been designed to cleanse, relieve irritation or itching and decrease odour. Such messaging reinforces VP as ‘normative’ and ignores the evidence that the vagina is self-cleansing.^37^

The reporting of VP decreased over time during the study despite the absence of a structured, standardised intervention. While this may have been because of social reporting bias, another possible explanation may be the basic education provided by clinicians who routinely informed participants engaging in VP that the vagina is self-cleansing and that VP could harm the vaginal mucosa leading to increased risk for infections. They also cautioned participants against self-medicating perceived offensive odours as these could indicate GTIs requiring prescription medication. The only other study in South African women that assessed VP over time showed only a 10% decrease in VP during the study despite VP being discouraged as part of counselling at each quarterly study visit.^30^ Because reduction in VP was less likely in women in the intervention arm, which included a diaphragm and microbicide gel, the authors hypothesised that the reason for the difference between the arms may have been because of the manual insertion and removal of the diaphragm, increased post-coital discharge from the gel or perceived cleansing properties of the gel. In Zambia, an interventional study to decrease intra-VP in women living with HIV was unable to show significant decline after 8 weeks in either the control (a 3–5-min brief message advising women not to engage in VP) or intervention (a 20–30-min socio-educational intervention) groups.^35^ In our study, visits were more frequent, occurring 1–4 weeks apart, perhaps indicating that to meaningfully reduce these culturally ingrained, normative practices, frequent and consistent messaging is needed.

Young women who receive positive GTI test results might be motivated to change their vaginal hygiene practices, particularly when education has been provided about the link between VP and GTIs; however in our study, laboratory-confirmed GTIs at enrolment were not associated with VP over time. There was a significant difference in the prevalence of laboratory-confirmed GTIs at enrolment compared to reported symptomatic infections This supports data from other South African cohorts, highlighting the urgency to introduce routine laboratory screening for GTIs into primary healthcare settings.^38,39^

Studies show that women use VP as a means to reduce GTI risk.^25^ We hypothesised that women would be more likely to engage in VP and use condoms consistently with partners who they thought posed a greater risk for GTIs, such as casual partners, transactional partners or partners who they knew had other partners. However, despite the high prevalence of casual sexual partnerships, self-reported transactional sex with casual partners and casual or new partners with concurrent partners, condom use was poor with all partner types. We also found that women who used condoms inconsistently and where last sex was with a casual partner were 3 times more likely to report VP. It is possible that these women used VP to compensate for unprotected sex with partners that they perceived as posing a higher risk of GTIs. The use of VP after higher risk sex emphasises the need to include VP during risk reduction counselling.

Our study has limitations. HVTN 915 was 3 months long, so the sustainability of the reduction in VP could not be assessed. Data were collected through an interviewer-administered questionnaire, which may have introduced social desirability bias in the self-report of study information. The interview technique of the interviewers may have biased the data. Although there may have been some recall bias at the screening assessment, the 7-day recall period thereafter would have reduced this during the study. The content of the education provided on reducing VP was not required to be documented, and thus the consistency of the messaging about avoidance of VP cannot be proven.

In the context of the disproportionate risk of HIV in young South African women, further interventions are required to modify VP. We strongly recommend the development and evaluation of interventions, such as the use of simple educational messages during HIV risk reduction counselling, that may be beneficial to reduce the added risks of VP on HIV acquisition.
